# Production of Œdema

**Published:** 1870-12

**Authors:** 


### Production of Œdema.

We suppose that if any of our readers were asked what would be the effect of suddenly applying a ligature to the principal vein of a limb, or in any other way arresting the return of the blood through it, the immediate answer would be, that congestion would occur, which would relieve itself by serous exudation through the coats of the vessels; or, in other words, that œdema of the whole limb below the point at which the circulation was arrested would take place. If, however, it were further urged that cases daily come under observation in which particular veins have become obliterated at the pressure of tumors or what not, and yet that such obliteration is not followed by any œdema or dropsical accumulation, the stereotyped reply would be, that in such cases the retardation of the blood current had occurred so slowly that sufficient time had elapsed to enable the collateral channels to become dilated, and to convey by a thousand smaller vessels the blood which was previously transmitted by one. At a recent *seance* of the Academie des Sciences, however, M. Ranvier adduced certain experiments which, if they do not absolutely disprove the ordinarily received views, at least are strongly suggestive of the suspicion with which we should regard all traditional dogmas, however high the authority by which they are supported. The views above-mentioned seem to date from the experiments made by our countryman, Richard Lower, who, in his "Essay on the Heart and on the Colour and Movement of the Blood," first showed that tying the vena cava was followed by ascites, and ligature of the jugular veins by œdema of the head, with copious flow of saliva and tears, resembling, as he says, the salivation produced by mercury, terminating in two days in suffocation. Although apparently conclusive, these experiments were not universally accepted, and even so recent an observer as Hodgson, states that he had seen several instances in which the femoral vein was obliterated, and one in which it was included in a ligature, without any unfavorable consequence. In 1823, M. Bouillard, in an important *memoire* that was published in the *Archives Generales de Medicine,* again took up the views of Lower, and corroborated them by the details of cases in which, when œdema affected a certain portion of the body, he found the corresponding vein obliterated either by a tumor or by a clot which had formed after delivery. From the period when this memoir appeared the general impression has been that the obliteration of the principal vein of a part sufficiently accounts for œ ema into its tissue. M. Ranvier, however, appears to have been dissatisfied with the accepted views on the subject, and proceeded to repeat the second experiment of Lower. He tied the two jugular veins at the inferior part of the neck in a dog and in a rabbit. To his surprise, however, these animals presented no discharge of tears, no salivation, nor any œdema of the head. In other experiments he ligatured the femoral vein immediately below the crural ring in the dog; but here again no œdema occurred either on the day of operation or at any subsequent period. These results, consequently, were in accordance with those observed by Hodgson in man. Lastly, he applied the ligature to the inferior vena cava, but still no œdema occurred. He then conceived the idea of favoring the production of dropsy by paralyzing the vasomotor nerves, and, recalling the experiments and observations of M. Claude Bernard, he divided the sciatic nerve on one side in a dog, whose vena cava inferior had previously been tied. On this side a considerable degree of œdema immediately supervened whilst the opposite hind limb remained in its ordinary condition. This remarkable experiment was performed three times, and on each occasion with the same results. From these experiments M. Ranvier believes that he is justified in concluding that mere ligature of the vein does not, in the dog at least, produce œdema; but that after obliteration of the veins, dropsy may be caused by section of the vaso-motor nerves. The same probably holds good in the case of man, and it is easy to comprehend how important are the practical results that may follow the application of this view.—*Lancet.—Jour. Cutaneous Med.*

